# Surface modifications for phase change cooling applications via crenarchaeon *Sulfolobus solfataricus* P2 bio-coatings

**DOI:** 10.1038/s41598-017-18192-2

**Published:** 2017-12-20

**Authors:** Ahmad Reza Motezakker, Abdolali Khalili Sadaghiani, Yunus Akkoc, Sorour Semsari Parapari, Devrim Gözüaçık, Ali Koşar

**Affiliations:** 1 Faculty of Engineering and Natural Sciences, Sabanci University, Istanbul, Turkey; 2 Sabanci University Nanotechnology and Application Center (SUNUM), Sabanci University, Istanbul, Turkey; 3 Center of Excellence for Functional Surfaces and Interfaces for Nanodiagnostics (EFSUN), Sabanci University, Istanbul, Turkey

## Abstract

Due to its high heat removal capability and exploitation of latent heat, boiling is considered to be one of the most effective cooling methods in industry. Surface structure and wettability are two factors imposing boiling phenomena. Here, we propose an effective and facile method for surface enhancement via crenarchaeon *Sulfolobus Solfataricus* P2 bio-coatings. The positive effects of such surfaces of bio-coatings were assessed, and enhancements in heat transfer and cooling were obtained. Visualization was also performed, and bubble dynamics of generated bubbles and vapor columns from the tested surfaces with bio-coatings are here presented. Superior performance in terms of boiling heat transfer and cooling was reached with the use of crenarchaeon *Sulfolobus Solfataricus* P2 coated surfaces. Thus, this study clearly demonstrates the potential of futuristic surfaces with bio-coatings to achieve substantial energy saving and efficiency.

## Introduction

**Figure 1:**
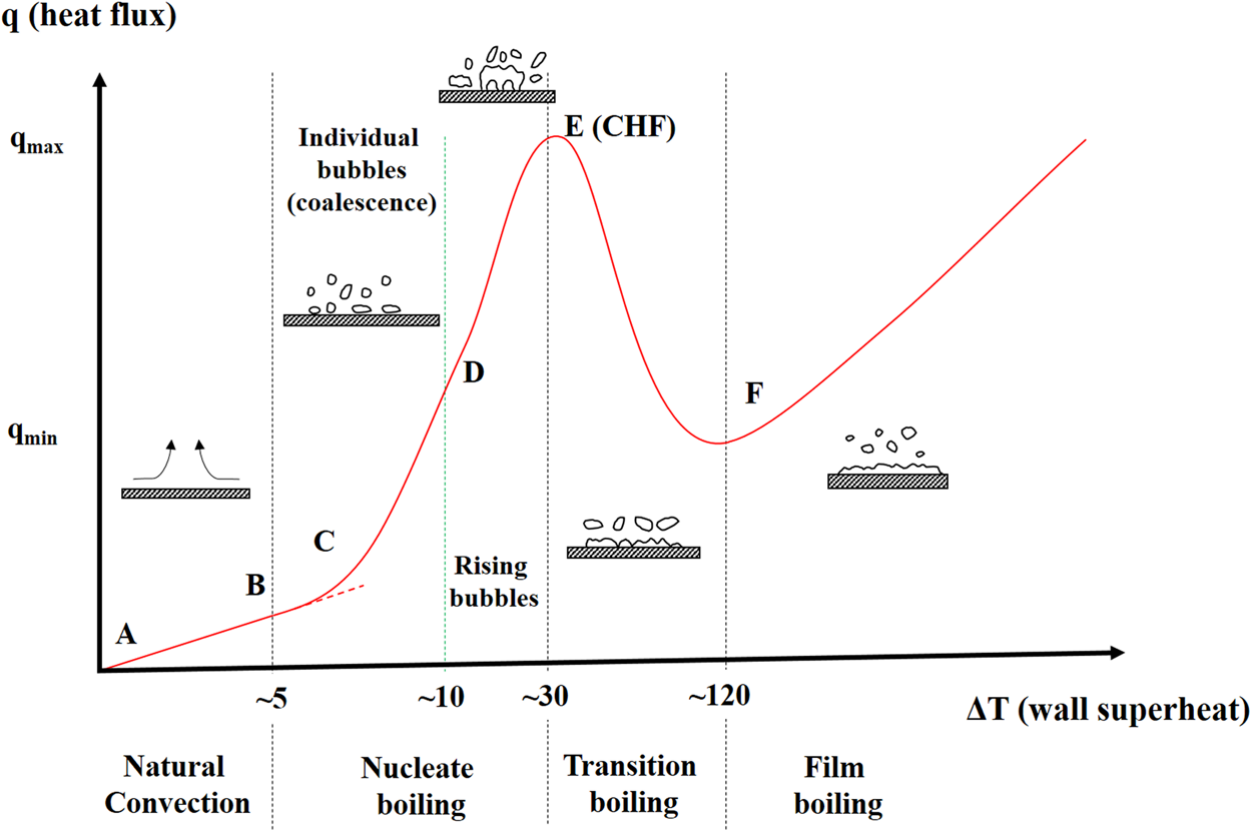
A typical boiling curve for pool boiling including natural convection, nucleate boiling, transition boiling and film boiling. A-B: single phase natural and later forced convective heat transfer from the heater surface to the liquid. B-C: the slope of heat flux-wall temperature increases marking nucleate boiling. The first isolated bubbles can be seen on the hot surface at point C. C-D: individual bubbles emerge and depart from the surface. D-E: in this region, the rate of bubble generation increases rapidly, which results in interactions among adjacent bubbles. More lateral coalescence of vapor columns contributes to the formations of dry spots. The fully developed nucleate boiling region ends with the critical heat flux condition (point E, CHF). E-F: beyond CHF high vapor generation causes a vapor blanket and a dramatic increase in the surface temperature leading to the burnout condition.

Due to the potential for obtaining high heat removal rates with boiling phenomena, many applications related to electronic cooling, power generation, refrigeration and distillation involve phase change heat transfer^1^. As a result, many recent studies have been conducted to enhance boiling heat transfer and reach ultra-high heat flux cooling. When a typical boiling curve is examined, there exist single phase natural and later forced convective heat transfer mechanisms prior to nucleation from the heater surface to the working liquid (Fig. 1 point A to B). As the heat flux increases, nucleation boiling incepts, and isolated bubbles can be first seen on the heated surface (Fig. 1 point C). In this region, the bubble departure frequency and number of active nucleation sites are dependent on the thermal boundary conditions (wall superheat, wall heat flux, surface morphology) (Fig. 1 point C to D). In the fully developed nucleate boiling region, the rate of bubble generation increases rapidly, thereby resulting in interactions among adjacent bubbles and generating vapor columns on the surface (Fig. 1 point D to E). For higher wall superheat, a greater lateral coalescence of vapor columns contributes to the formation of dry spots. The maximum in the profile highlights the critical heat flux (CHF) condition (Fig. 1 point E, corresponding to CHF). Beyond this point, high vapor generation causes a vapor blanket, which covers the surface. Consequently, the vapor blanket acts as an insulating layer and leads to a dramatic increase in the surface temperature, resulting in burn-out condition on surfaces (Fig. 1 point E to F). Due to its dependency on many parameters, the CHF phenomenon is complex and hard to predict. Kutateladze^2^ and Zuber^3^ proposed correlations for CHF, while only considering properties such as working fluid, gravity and system pressure. Later researchers reported that nucleate boiling heat transfer and CHF are both significantly affected by surface roughness^4,5^, wettability^6,7^ and heater properties such as size and orientation^8^. There have been many studies on the enhancement of boiling heat transfer and widening of the safe working condition range of thermal systems via surface modifications^9–11^. Recently, the effects of textured surfaces such as nanowire arrays^12,13^, porous media^14,15^ and graphene structures^16^ on boiling heat transfer and CHF have been investigated in the literature.

The combination of porosity and hydrophilicity provides the greatest enhancement in CHF^17^. Porous layers mostly enhance CHF by providing vapor escape paths^18,19^. According to reported data^20^, thick porous layers do not perform well in terms of heat transfer at high heat fluxes due to higher number of dry spots.

Here, we propose a novel bio-coating for enhancing heat transfer and CHF in pool boiling via *Sulfolobus Solfataricus* P2, which is a thermophilic archaeon. Nowadays, they are known to be a large and diverse group of organisms which are widely distributed in nature and are common in all habitats^21^. They are divided into five phyla^22,23^. Archaeon cells have characteristics similar to those of eubacteria, including unicellular morphology. They have a circular chromosome and resemble eukaryotic cells due to their metabolisms involving DNA replication and transcription^24,25^. Most strikingly, archeal cells have unique habits to keep themselves alive under physiologically harsh conditions such as low or high temperatures (−2 °C to 15 °C or 60 °C to 122 °C), high salinity (2 M to 5 M NaCl), and low or high pH (<4 or >9)^26–28^. The hyperthermophilic archaeon called *Sulfolobus Solfataricus* belongs to the Crenarchaeota phylum. It was first isolated by Pisciarelli Solfatara in Italy^29^. *Sulfolobus Solfataricus* is an irregular and lobe-shaped archaeon with a size in the range of 0.2 to 2 µm which grows optimally at 80–85 °C, has a pH of around 3 (while maintaining intracellular pH around 6.5), and can utilize variable carbon sources to maintain cellular homeostasis^30^. The proposed bio-coating is environmentally friendly and is known to be a large and diverse group of organisms. It can endure severe environmental conditions. In addition to abundancy of the *Sulfolobus Solfataricus* P2, the coating process is cheap and fast compared to other bio-coating processes. The coating process was optimized before performing the boiling experiments. It is known that heating temperature and evaporation time are two main factors affecting the cure heat process. Therefore; different configurations were examined to optimize the coating method. Using this coating method, it is possible to soak the interested part of device with an arbitrary geometry into the mixture and obtain the desired coating thickness. In this study, we propose a new bio-coating, which is highly durable, environmental friendly, cheap and has a practical coating method and unique structure, which makes the proposed bio-coating a remarkable candidate for heat transfer applications. Thus, this robust and heat resistant microorganism is a good candidate for providing organic porous coatings for energy saving and efficiency in an economical, facile and environmentally friendly fashion, which constitutes the motivation behind this method.

To investigate the effect of thermophilic crenarchaeon bio-coatings on boiling heat transfer and CHF, pool boiling experiments were conducted on silicon surfaces which were coated with different concentrations of thermophilic archaeon. Two solutions of Poly-L-lysine to archaeon with ratios of 1:2.5 and 1:5 were used to coat silicon samples, which led to archaeon layer thicknesses of 1 and 2 µm. All the experiments were conducted with deionized water under atmospheric conditions. The test area (sample size) was as 1.5 × 1.5 cm^2^.

## **R**esults

### Sample preparation and characterization

**Figure 2:**
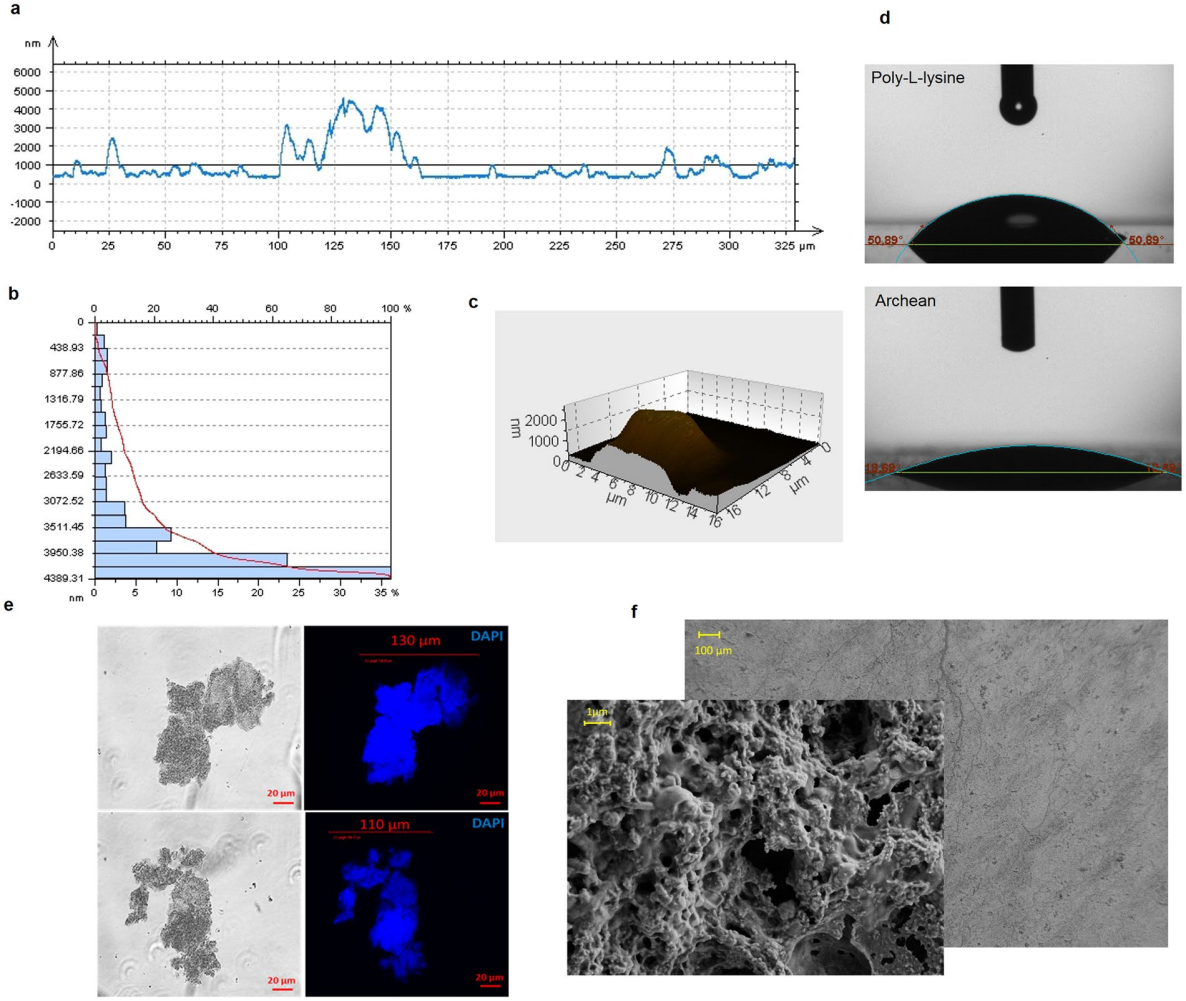
Samples were characterized before and after each experiment using the scanning electron microscope, Profilometery and contact angle measurement techniques and Fluorescence micrograph of cellular structure from archaeon (**a**) 2-D surface profile showing the cavities size and shape on the tested samples (**b**) Cavity size distribution showing the distribution of the cavities on the tested samples. (**c**) 3-D surface profile of a cavity with ~2 µm (**d**) water contact angle measurement on silicon surfaces and 1 µm crenarchaeon coated samples (**e**) Fluorescence micrograph of cellular structure from archaeon. DNA stained by DAPI (Blue) (**f**) SEM images of coated surface showing surface porosity.

All the information about crenarchaeon *Sulfolobus Solfataricus* P2 preparation and the coating process is mentioned in Online Methods and Supplementary Figure 1. The bio-coated surfaces were produced on 500 µm thick silicon wafer substrates with the heat cure method, which is easier and less expensive than other coating methods. In order to ensure the repeatability of the examined surfaces, the samples were characterized before and after each experiment. The coated surface structures were analyzed and characterized using Scanning Electron Microscope (SEM) techniques. Cavity size, shape, range and distribution along the surfaces were obtained using the 2-D and 3-D surface profilometer technique. Furthermore, surface wettability was measured with the contact angle measurement technique. Figure 2a,b and c show the 2-D surface profile, cavity size distribution, and 3-D surface profile of the coated sample with the thickness of 2 µm, respectively. As can be seen, large colonies with structures of a minimum height of 1 µm are located separately with distances between them up to 100 µm, which creates a nano-micro interconnected porous medium (Supplementary Figure 2). This medium can also be observed in the SEM image (Fig. 2f). The cavity size distribution (Fig. 2b) indicates that more than 60 percent of the surface is coated with large colonies with sizes bigger than 4 µm. The static contact angle measurements of the silicon wafer and bio-coated silicon wafer are presented in Fig. 2d, which shows that surfaces with bio-coatings have a higher wettability. On the other hand, dynamic contact angle measurements on the bare, 1 µm, and 2 µm coated surfaces have receding contact angles of ~51°, ~18°, and ~15°, respectively. This highlights the pronounced wetting behavior of the coated surfaces in boiling, especially prior to the critical heat flux condition. The porous nano-micro structures provided by such bio-coatings offer separate channels to intensify wicking flows through the media. The interconnected porous media are responsible for the lower contact angle followed by higher surface wettability, which also generates capillary motion within pores in such structures. This observation agrees with the reported data reported by Singh *et al*.^31^ and Das *et al*.^32^.

### Experimental setup and test section

**Figure 3:**
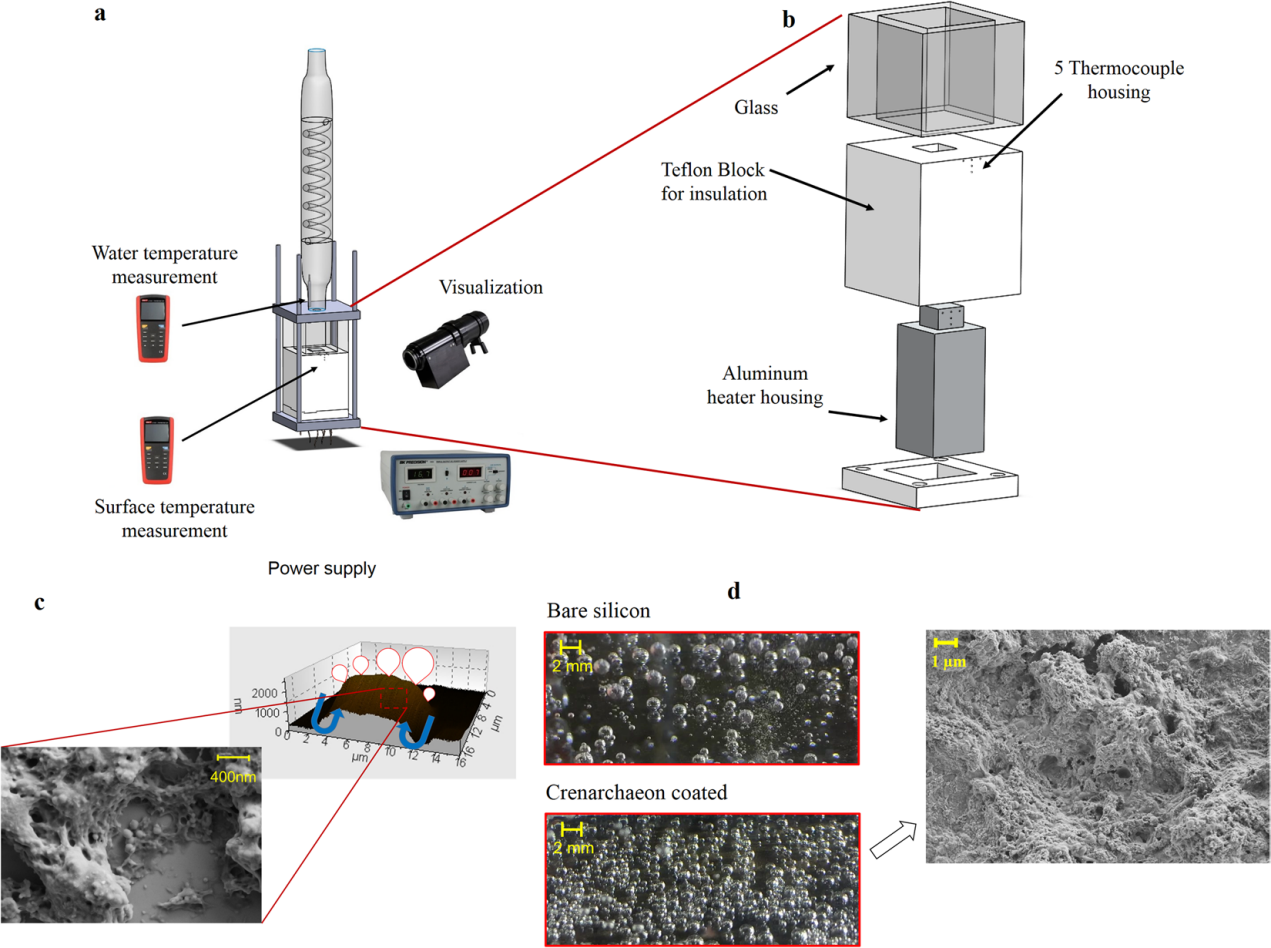
Schematic of the experimental setup, porous structure, wicking flow and vapor escaping. The experimental setup consists of test section, power supply for bottom wall heating, a condenser test section and high speed camera. Cartridge heaters were heated using the power supply. A Teflon block was used to decrease the amount of heat loss to the environment. 5 thermocouples were used (**a**) Experimental setup (**b**) Test section (**c**) porous surface, wicking flow and vapor escape (**d**) Nucleation site density of silicone surface and crenarchaeon coated surface.

The schematic of the experimental setup is displayed in Fig. 3a and b. The glass block is a hollow cube and has outer and inner dimensions of 60 × 60 × 60 and 40 × 40 × 40 mm^3^, respectively. The aluminium heating part has four vertical housing holes at the bottom for cartridges and five holes for T-type thermocouples (Supplementary Figure 3). With the help of a digital power supply with high precision multimeters, current and voltage were adjusted. The power supply was connected to cartridge heaters, which were press-fitted into cylindrical holes, while high quality conductive silicon grease was utilized to fill the gap between the cartridge heaters and inner areas of the holes. Two holder plates were used to sandwich the glass block and the Teflon block. The upper holder plate has four holes, which were used to fill up the glass block with working fluid, insert a thermocouple to measure bulk temperature of fluid, insert a vertical heater to keep the fluid at saturation temperature and make a connection with the vertical condenser to provide a constant supply of deionized water.

Plastic gasket sealers with resistance to high temperatures were used between glass block edges and upper plates to prevent any leakage. The reflux condenser is made of concentric glass tubes of inner and outer diameters of 22 mm and 40 mm, respectively, and a length of 40 cm. The gap between the outer and inner tubes was filled with water to condense the vapor escaping through the inner tube, which is open to atmosphere, to maintain the tests at the ambient pressure. The volume of liquid was measured before and after each test to check for the change in the liquid amount. It was found that the vertical reflux condenser was efficient, and the amount of water remained nearly the same.

All the temperatures and power readings were recorded under steady state conditions. To ensure repeatability, every sample was tested three times. The heat flux was increased in small steps until the CHF point was reached. At this point, an excessive rise in wall temperature and a vapor blanket on the samples were observed. The experimental data were reduced to obtain the heat transfer coefficient and heat flux.

A high-speed camera (250 frames/sec) was used to visualize pool boiling experiments. Bubble dynamics and behavior prior to and during the departure were examined and analyzed to attain a better understanding of the enhancement mechanism. For each experiment, more than 50 bubbles were selected to determine average bubble departure volume.

### Heat transfer performance

**Figure 4:**
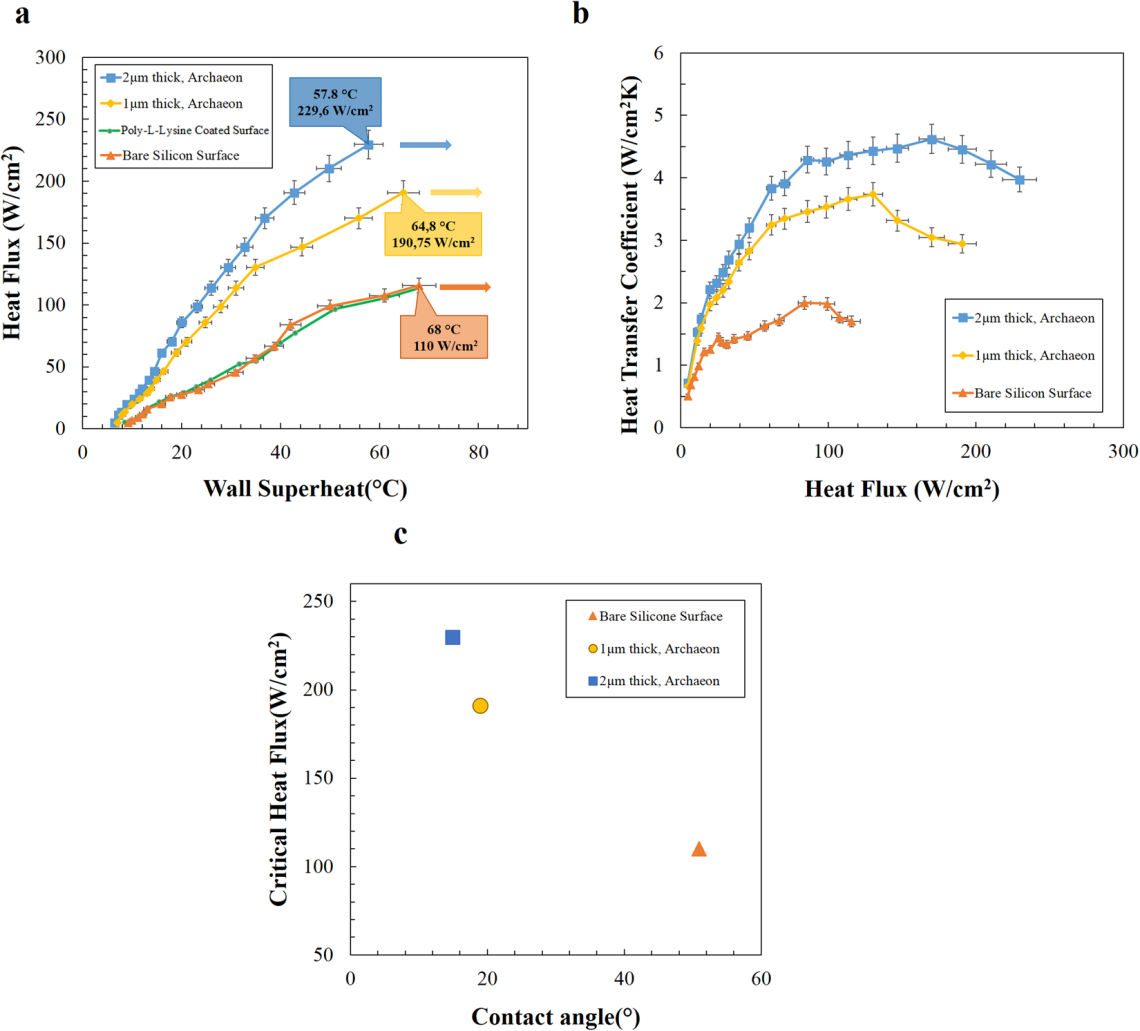
Heat transfer coefficients for coated and plain surfaces. The effect of coating thickness on heat transfer performance of the surfaces was obtained using applied wall heat flux, wall and fluid temperatures. Critical heat fluxes and enhancements via bio-coated surfaces are presented. (**a**) wall superheat-heat flux profile (**b**) heat flux-heat transfer coefficients profile (**c**) obtained critical heat flux values as a function of surface contact angle.

Before conducting pool boiling experiments on bio-coated surfaces, the independency of the results from the Poly-L-lysine adhesive layer was tested (as stated in the sample preparation section, Poly-L-lysine was used for transferring crenarchaeon coatings on the silicon surfaces). By comparing the obtained results from the bare silicon surface and the Poly-L-lysine coated silicon surface (Fig. 4a), it seen that the adhesive layer had no effect on pool boiling heat transfer.

Crenarchaeon coatings with two different thicknesses were used to investigate the effect of the proposed bio-coating thicknesses on the pool boiling heat transfer performance. Figure 4a shows the obtained heat flux-wall superheat profiles for different examined surfaces. As can be seen, an increasing trend is observed for all the tested samples. The obtained results indicate that for a constant wall heat flux, significant lower wall temperatures were obtained on bio-coated surfaces (for both thicknesses) at the same heat flux compared to the uncoated samples. In other words, the rate of wall superheat increase is much lower for crenarchaeon coated surfaces relative to that of the bare and Poly-L-lysine coated surfaces. Furthermore, it can be observed that wall superheat increase decreases with coating thickness at a fixed wall heat flux.

The heat transfer coefficient (Supplementary Notes 1 and 2) profile is shown in Fig. 4b. At a fixed heat flux, heat transfer coefficients obtained from the crenarchaeon coated surfaces are higher than those of the bare silicon surface, while the heat transfer coefficient increases with the coating thickness. Maximum enhancements of 94.2% and 126.7% were obtained for 1 µm and 2 µm thick coatings, respectively, in comparison to the bare silicon surfaces.

The critical heat flux (CHF) location and corresponding wall superheat are labeled for each sample in Fig. 3a. As can be seen, for all the bare and crenarchaeon coated surfaces, the heat transfer coefficient increases with heat flux until the critical heat flux (CHF) point. According to the obtained results, the CHF of the bare silicon surface is measured as 115 W/cm^2^ (corresponding wall superheat of 68 °C), while CHF values of bio-coated surfaces reach 190.75 (corresponding wall superheat of 64.8 °C) and 229.16 W/cm^2^ (corresponding wall superheat of 57.8 °C) for surfaces with coating thicknesses of 1 µm and 2 µm, respectively. It can also be observed that CHF increases with coating thickness.

## Discussion

Surface modification is one of the most used techniques for heat transfer and critical heat flux enhancement in heat transfer applications involving phase change. Heat transfer results indicated that the crenarchaeon and their aggregates form a bio-coating on the surface, which changes the surface structure for better heat removal. While chemical surface treatments are severely toxic, such bio-coatings are suitable candidates to change the surface structure in an environmental friendly and biocompatible fashion.

Interactions between surfaces and organisms were investigated and different biological structures such as complex microbial communities (bio-films) were reported. Studies revealed that archaeal species adhere to either biotic or abiotic surfaces, and they are able to form multicellular complex bio-film structures under a wide range of extreme environmental conditions including hydrothermal vents, under water springs of the Dead sea or on walls of sulfide-rich cave system together with bacteria^33–36^. Cellular aggregation and proliferation mediated by flagella or type IV pili maintain the mature architecture of these structures^37,38^. Furthermore, they were also embedded in a mesh of proteins, polysaccharides and lipids called EPSs (extracellular polymeric substances), which can further modify the surface^39,40^.

*Sulfolobus* species can also form different types of architectures on the surface. *Sulfolobus Solfataricus* species form flat bio-films with low cell density, while *Sulfolobus acidocaldarius* species lead *to* bio-films with tower-like aggregates^41^. The archaeal pili of *Sulfolobus Solfataricus* are found to mediate surface adhesion^40^. Moreover, mutant strains showed the importance of the pili function and the structure for the architecture^40,42,43^. The studies reported that the amount and composition of the EPSs are involved in the formation of the architecture, and the first enzyme, which affects EPSs, is identified in *Sulfolobus Solfataricus* ^44^. In addition, environmental conditions including temperature, pH, salt concentration and exposure to UV can modify the archaeal communication and ultimately affect the surface structure covered with crenarchaeon^43,45^. Our results support that monoculture of the crenarchaeon can lead to flat structures through mimicking natural conditions.

**Figure 5:**
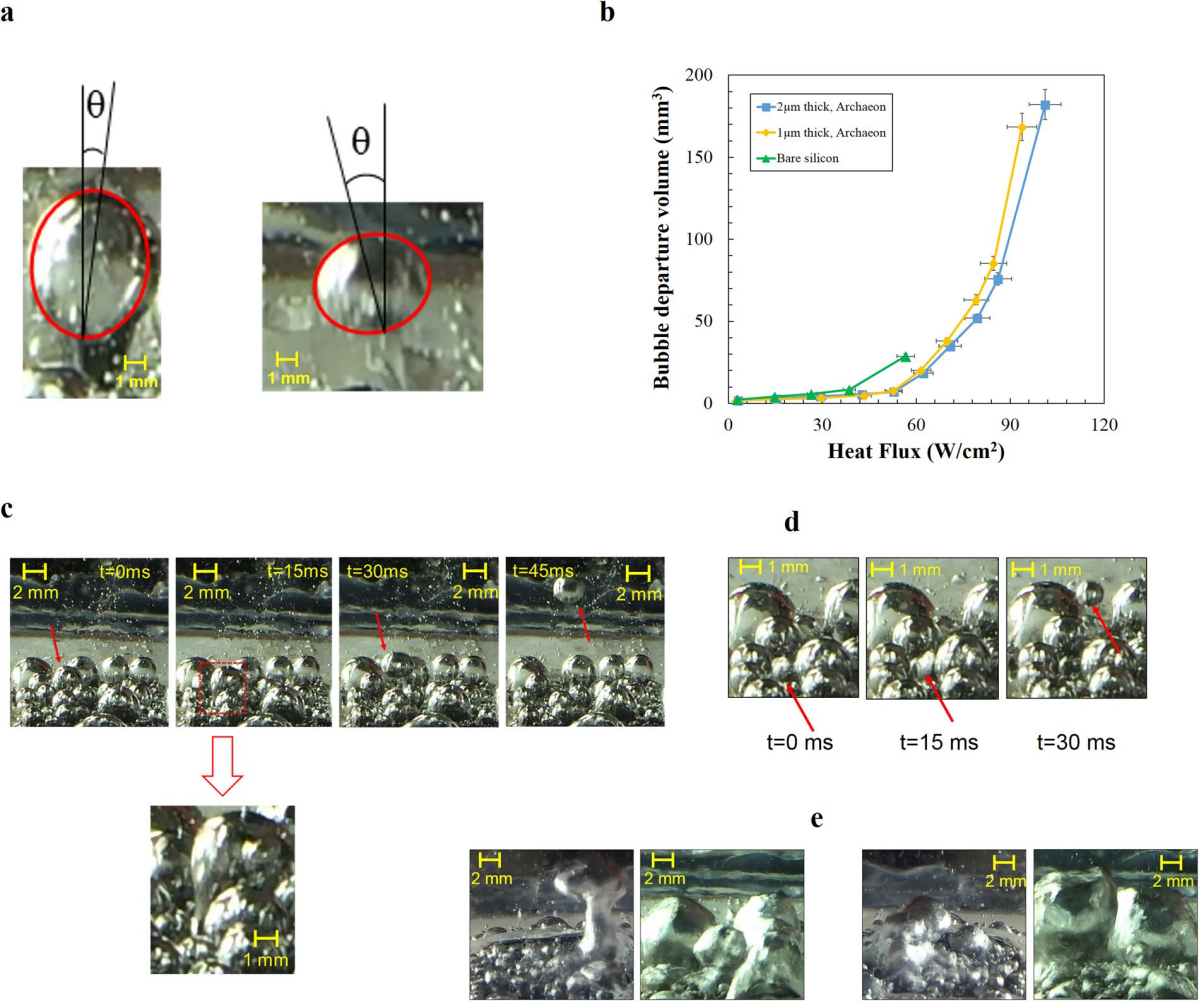
Boiling images from coated and uncoated surfaces. Visual results were obtained using the high speed camera system. Bubble dynamics and vapor columns were investigated in order to have an understanding about the enhancement mechanisms (**a**) bubble images on coated surfaces at 50 W/cm^2^. Shape of bubbles is ellipsoid according to the images (**b**) bubble departure volume of bare silicon (green line), 1 µm thick (yellow line) and 2 µm thick (blue line) bio-coated surfaces (**c**) inclined departed bubble (**d**) isolated bubble in nucleate boiling region (**e**) vapor columns on uncoated and coated surfaces.

The significant enhancements in both CHF and boiling heat transfer with crenarchaeon coated surfaces are attributed to the physical structure of coated surfaces. As shown in Fig. 3d, the coated layer creates a porous structure with numerous pores, which act as nucleation sites during boiling. The size of pores ranges from 100 nm to 2 µm. Therefore, the aggregation of these pores produces more nucleation sites on the porous coated layer and result in higher heat transfer rate^46^. This can be clearly examined in Fig. 5 showing more active nucleate sites on the coated surface compared to the bare silicon surface. The existence of a porous layer has a great effect on liquid transportation inside the structure. There are many interconnected porous channels, which aid liquid transportation between the pores beneath the surface resulting in the CHF delay^46^. Capillary pumping is another mechanism, which has a considerable effect on the surface rewetting by providing liquid flow to dry spots. In other words, capillary flow reduces liquid-vapor counter flow resistance by providing flow path for liquid and vapor and prevents the dry-out condition^47^. In the literature^48^, a method was reported to release trapped vapor in a porous layer by adding vapor channel, which offers paths for escaping vapor. It can thus be hypothesized that the colonial structure of archaeon porous layer can provide separate vapor channels through the porous layer. These channels can release trapped vapor at the bottom of the porous layer, thereby delaying the CHF condition. Electron microscopy and surface profile images (Fig. 2a,b,c and f) indicate that the crenarchaeon coatings are distributed over the surface like separated colonies with a minimum size of 1um. The distance between these colonies are coated with layers of archaeon acting as channels for vapor venting inside the porous layers. These channels not only remove the vapor layer at the bottom of porous structure but also separate the vapor and liquid path flow, which is another reason for CHF and HTC enhancement^48^.

The effect of bio-coatings was also examined using images from the high-speed camera (250 frames/sec). Since it was difficult to define the average bubble diameter at high heat fluxes, the bubble departure volume is considered instead of the average bubble departure diameter. Most bubbles are not spherical except at low heat flux (<60 W/cm^2^). Therefore, we assume that all bubbles were ellipsoid, as shown in Fig. 5a. The volume shrinks with the coating thickness. With bio-coatings, bubble release frequency (~7.5 Hz, ~33 Hz, and ~38 Hz for silicon, 1 µm archaeon and 2 µm archaeon coated surfaces, respectively, at the heat flux of 56 W/cm^2^) increases accompanied with the decrease in the bubble volume, which serves as a proof for the performance and energy efficiency enhancement in phase change systems (Supplementary Note 3 and Supplementary Figure 4).

In conclusion, we propose crenarchaeon *Sulfolobus Solfataricus* P2 bio-coatings for performance enhancement. The novelty of this study lies in the new type of bio-coating and heat transfer enhancements in nucleate boiling due to its surface morphology. These bio-coatings offer enhanced performance and have the potential for addressing high heat removal requirements in many applications including heating and cooling devices, thermofluidic systems, batteries and microfluidic and nanofluidic devices.

## Methods

### Sample preparation

*Sulfolobus Solfataricus* is one of the hyperthermophilic and acidophilic archea. It belongs to *Sulfolobus* species and could be a good model for temperature dependent phenomena such as cooling.

For this purpose, *Sulfolobus Solfataricus* P2, was grown at 80 °C, pH of 3 in a batch culture under mild agitation. Basal salt medium, Brock modified Allen medium^37^ were used to obtain optimal growth. However, as the salt medium is based on minimal media containing only minerals as a carbon source, we supplemented the basal medium with different concentrations of Sucrose (0.5, 2, 5 g/L) with 0,2% (w/v) Tryptone. Stock cultures were maintained in 2 g sucrose/L and 100 g glycerol/L. Cultures were started from −80 °C stock; cells were inoculated into 50 mL fresh culture medium. After 24 h of propagation, the cell culture was transferred to 500 mL of the pre-heated new medium. Cell growth was then monitored with UV Spectrophotometer at 600 nm following each 24 h till 96 h. 1 ml of cells were pelleted at 4000 g for 5 min and then re-suspended in 500 μl of 4% (v/v) PFA for 20 min and then permeabilized with 0.1% (v/v) Triton X-100 at RT for 5 min. Fixed and permeabilized cells were washed with PBS buffer twice and then cells were spread on Poly-L-lysine coated cover slide. After air-drying, cells were stained with DAPI (4’,6-diamidino-2-phenylindole, 10 μM). Then, coverslides were mounted and inspected under 40X magnification using a BX60 fluorescence microscope (Olympus, BX60, Japan).

Archaeon culture (OD 600 = 1, after almost 72 h later) was cooled down on ice, then centrifuged for 15 min at 4000 g and washed twice with ice cold phosphate buffer. Pellet was then resuspended in 5 ml PBS (0.1 g/ml), and 2.5 ml of this solution was mixed with 1 ml Poly-L-Lysine (0,01% (w/v) in H20) to cover 500 µ thickness silicon wafer substrate with heat cure method.

Crenarchaeon were cultured at pH 3 and 80 °C in a minimum salt solution including different amounts of sucrose (0.5, 2, 5 g/L). Time dependent growth was analyzed based on optical density at wavelength of 600 nm by spectrophotometer (Supplementary Figure 2). We obtained a more stable growth in the medium, which was supplemented with 2 g/L sucrose, and we performed all the experiments under the same growth conditions. Sulfolobus species have been known to form aggregates under certain conditions such as UV treatment^49^. We also wanted to analyze the growth and the status of the aggregates for better approaching to the surface structure. According to microscopy examination, we found that archaeon is found mostly at aggregated form (72 h, 2 g/L sucrose), and aggregates almost cover 20–30% of the surface (Fig. 1F).

### Sample characterization

A scanning electron microscope (FEG-SEM Leo Supra 35, Oberkochen, Germany) was used to obtain the microstructural images of the specimen surfaces before and after the treatment. SEM scans an electron beam on the surface of a specimen and measures a number of signals resulting from the interaction between the beam and specimen. One particularly useful imaging method is collecting low energy secondary electrons (SE) signals, which originate within a few nanometers from the specimen surface. Due to this process, SE method allows imaging of the surface with a high spatial resolution. The micrographs were collected using SE mode in low voltage (2 KV) within different tilts to allow a full imaging of the surface area and the cross sectional area. The wettability of bio-coated surfaces was tested by the WCA (water contact angle) method. The Sessile drop method was used to measure the CA (contact angle) by dispensing a 5 μL water drop, and the average CA from five different positions on each sample was taken into consideration. The dynamic contact angles were measured by holding the water drop with a stationary needle in contact with the surface and moving the goniometer stage along one direction. The surfaces were tilted at an angle of 25° relative to the horizontal direction. To check whether the angles are the true advancing and receding angles, the surface was further tilted to 50°. The angles remained nearly unchanged implying that these were representatives of the advancing and receding contact angles, respectively.

### Pool boiling experiments

As reported previously, for pool boiling experiments, we prepared the in-house made boiling set up. All the experiments were conducted at saturation temperature under ambient pressure with deionized water (DI) which was degassed by boiling for 15 min as working fluid. The water was heated to saturation temperature by an emerged cartridge heater. During the experiments, amount of working fluid was kept constant using a vertical condenser on top of the pool which was opened to atmospheric pressure. To decrease the heat loss, the aluminum heating part was insulated by a Teflon block. All the data was recorded under steady-state conditions, when the fluctuations in data from integrated sensors became negligible. The waiting time to reach the steady state condition depends on the heat flux. CHF condition was defined as the first detected excessive rise in wall temperature during the experiments. During the experiments, CHF corresponded to the heat flux, beyond which a meager increase in heat flux lead to a large surface temperature rise (more than 20 °C), and at which a thick vapor layer covered the heating surface. In most cases, CHF occurrence led to the burn-out condition.

## Electronic supplementary material

Supplementary Information
