# Supplementary material for: Surface modifications for phase change cooling applications via crenarchaeon Sulfolobus solfataricus P2 bio-coatings
Source: Sci Rep. 2017 Dec 20;7:17891. doi: 10.1038/s41598-017-18192-2 (PMC5738355; doi:10.1038/s41598-017-18192-2)
Supplement: Supplementary file 1 — Supplementary Information [file 41598_2017_18192_MOESM1_ESM.pdf]

Supplementary Information

# Surface modifications for phase change cooling applications via crenarchaeon *Sulfolobus solfataricus* P2 bio-coatings

Ahmad Reza Motezakker[1,2], Abdolali Khalili Sadaghiani[1,2], Yunus Akkoc[1], Sorour Semsari Parapari[1], Devrim Gözüaçık[1,3], Ali Koşar[1,3]

[1] Mechatronics Engineering, Faculty of Engineering and natural Sciences, Sabanci University, Istanbul, Turkey.
[2] Sabanci University Nanotechnology and Application Center, Sabanci University, Istanbul, Turkey
[3] Center of Excellence for Functional Surfaces and Interfaces for Nanodiagnostics, Sabanci University, Istanbul, Turkey

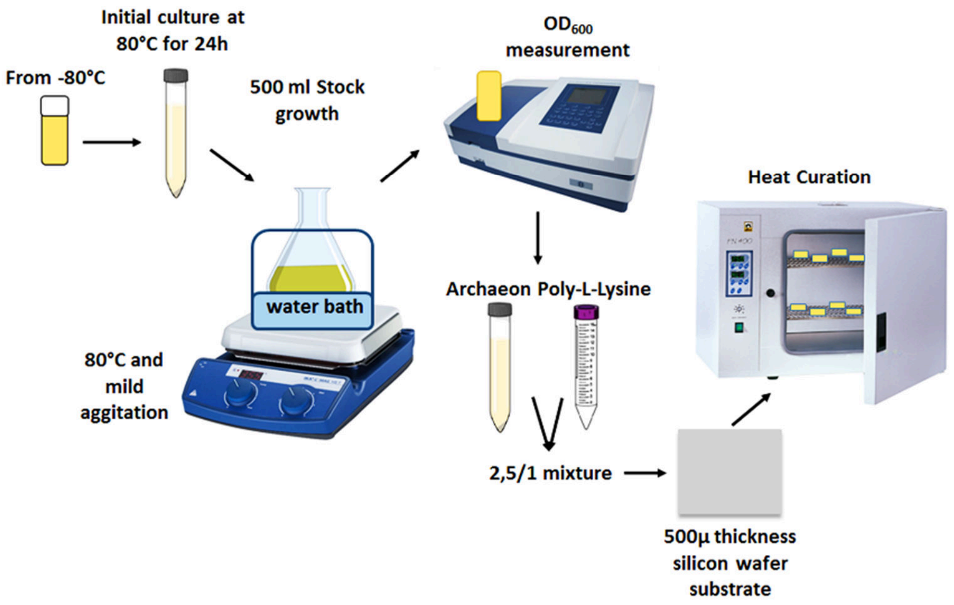


**Supplementary Figure 1**

**Schematic representation of the sample preparation**

*Sulfolobus solfataricus* P2, was grown at 80°C, pH of 3 in a batch culture under mild agitation. Cultures were started from −80 °C stock; cells were inoculated into 50 mL fresh culture medium. After 24 h of propagation, the cell culture was transferred to 500 mL of the pre-heated new medium. Cell growth was then monitored with UV Spectrophotometer at 600 nm following each 24 h till 96 h. Archaeon culture (OD 600=1, after almost 72 h later) was cooled down on ice, then centrifuged for 15 min at 4000 g and washed twice with ice cold phosphate buffer. Pellet was then resuspended in 5 ml PBS (0.1 g/ml), and 2.5 ml of this solution was mixed with 1 ml Poly-L-lysine (0,01 % (w/v) in H20) to cover 500µ thickness silicon wafer substrate with heat cure method. For this purpose, we used 60 °C incubator and we administrated the mixture on the silicon wafer substrate then waited until all liquid evaporate (after almost 30 min).

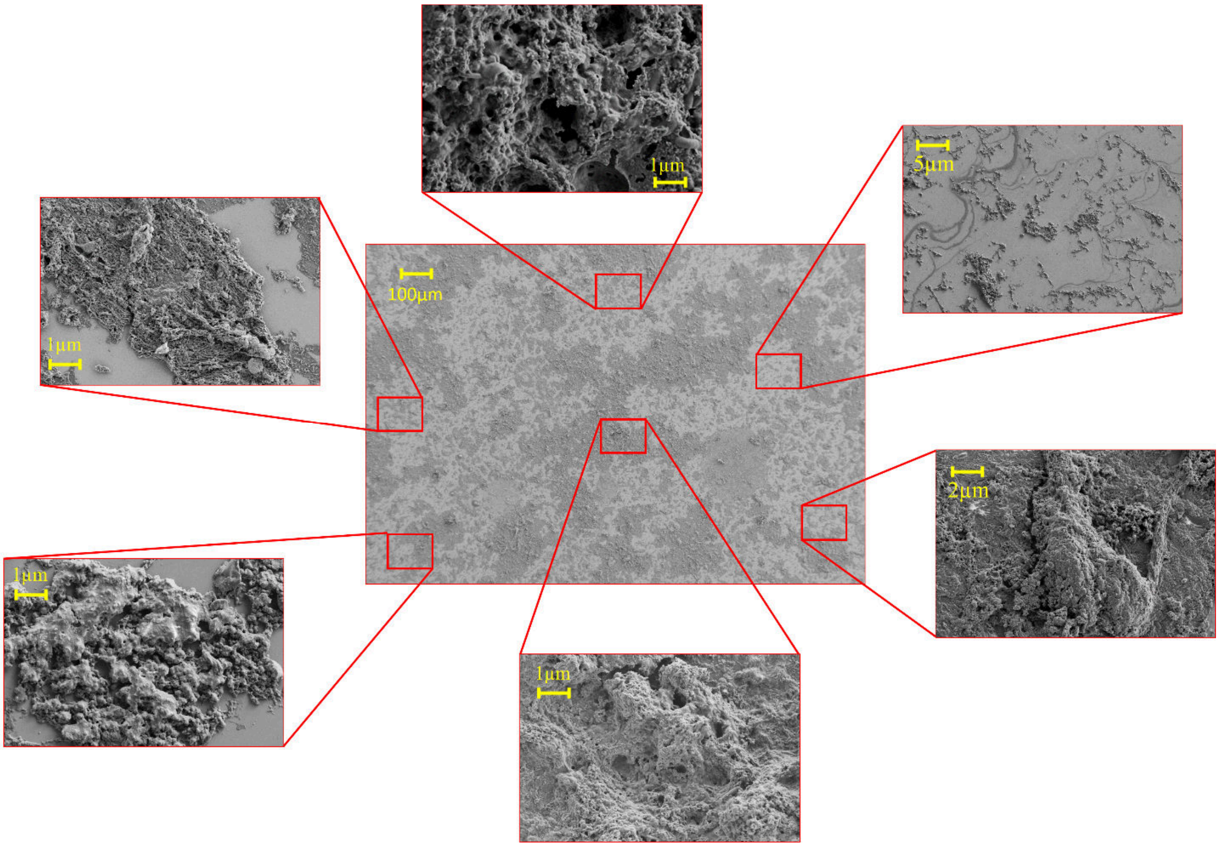

1µm
5µm
1µm
100µm
2µm
1µm
1µm

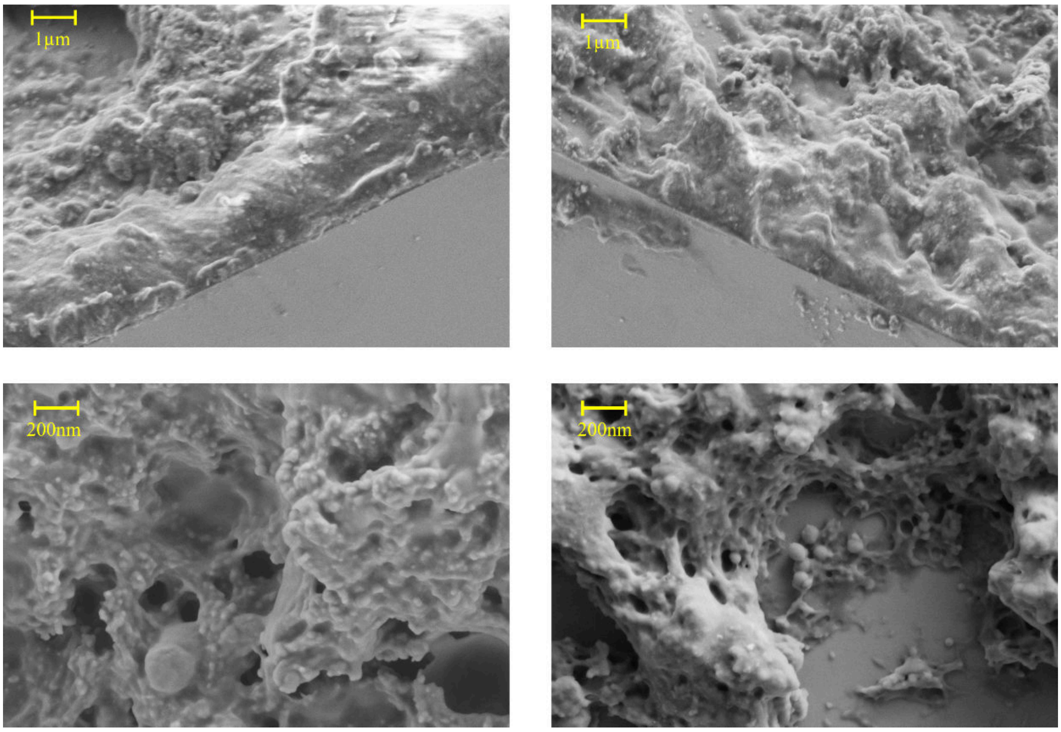


**Supplementary Figure 2**

Scanning electron microscopy images: Archaeon colonies with different thicknesses and shapes are shown in figure 2. A scanning electron microscope (FEG-SEM Leo Supra 35, Oberkochen, Germany) was used to obtain the microstructural images of the specimen surfaces before and after the treatment. SEM scans an electron beam on the surface of a specimen and measures a number of signals resulting from the interaction between the beam and specimen. One particularly useful imaging method is collecting low energy secondary electrons (SE) signals which originate within a few nanometers from the specimen surface. Due to this process, SE method allows imaging of the surface with a high spatial resolution[1]. The micrographs were collected using SE mode in low voltage (2 KV) within different tilts to allow a full imaging of the surface area and the cross sectional area.

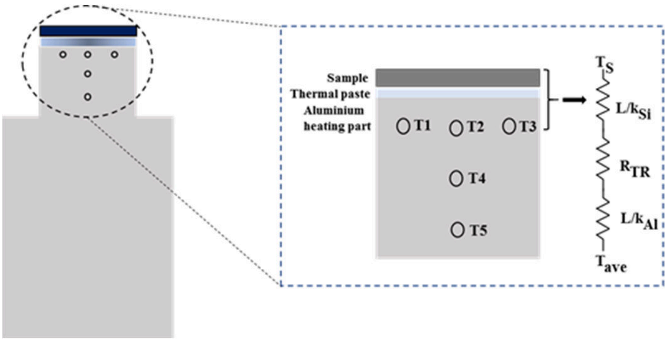


**Supplementary Figure 3**

Locations of the temperature measurements

There are five holes for thermocouples to read temperatures. The vertical temperature readings were used to obtain the vertical temperature gradient, while the horizontal temperature readings (located 1 mm beneath the sample) were used for wall temperature measurements. The surface temperature was calculated with the help of vertical measurements of $T_5$, $T_4$ and average temperature of the experimental setup right beneath the test section, $T_{ave} = (T_1+T_2+T_3)/3$. The surface temperatures are obtained by using the thermal contact resistance from the $T_{ave}$ to the silicon surface with the average of the thermocouple measurements. Five T-type thermocouples were used to record the temperatures.

Note 1. **Data reduction**

The net heat flux is calculated as follows:

$$q'' = \frac{VI - Q_{loss}}{A} \quad (1)$$

Here, V is the applied voltage, $I$ is the current, $Q_{loss}$ is the heat loss and $A$ is the heated surface area. Heat loss is the difference between input power and the amount of cooling energy in single-phase flow regime in boiling experiments. For minimizing the amount of heat loss, the aluminium heating part is surrounded by a Teflon block which is a prevalent as of insulator. To calculate the amount of heat loss for each test, a natural convection analysis was performed. The heat loss is expressed as:

$$Q_{loss} = VI - \dot{m} \times c_p \times \Delta T \quad (2)$$

Accordingly, the heat losses are less than 5%. The boiling heat transfer coefficient, $h$, is calculated as:

$$h = \frac{q''}{T_s - T_f} \quad (3)$$

where $T_s$ is the surface temperature, and $T_f$ is fluid bulk temperature. $T_f$ is measured using a thermocouple, while for saturated boiling the saturation temperature is considered as the saturated temperature at the local liquid pressure. The surface temperatures are obtained by using the thermal contact resistance from the $T_{ave}$ to the silicon surface with the average of the thermocouple measurements, as:

$$T_S = T_{ave} - q''\left(\frac{L_{Al}}{K_{Al}} + R_{TR} + \frac{L_{Si}}{K_{Si}}\right) \quad (4)$$

where $T_{ave} = \frac{T_1 + T_2 + T_3}{3}$ is the average temperature, $R_{TR} = 6\times10^{-6}\left(\mathrm{m^2K/W}\right)$ is the paste thermal resistance. The difference of saturation temperature, $T_{sat}$, and the surface temperature, $T_s$, is defined as the wall superheat $\Delta T_{sat}$.

Note 2. **Uncertainty analysis**

An uncertainty analysis is used for measuring instruments and experimental data based on the error propagation methodology proposed by Coleman and Steele [2]. The general formulation is expressed as:

$$U_y = \sqrt{\sum_{i=1}^{n} \left\{ \left( \frac{\partial y}{\partial x_i} \right) . U_{X_i} \right\}^2} \qquad (5)$$

where $U_{X_i}$ is the uncertainty in the parameter $X_i$. The calculated uncertainties are presented in **Supplementary Table 1**.

**Supplementary Table 1**

Uncertainties of experimental parameters

| **Parameters** | **Uncertainty** |
|---|---|
| Volt | ±1 V |
| Amper | ±0.1 A |
| Image pixel size | 250 µm |
| Wall Temperature | ±1-5% |
| Fluid Temperature | ±1-4% |
| Heat Transfer Coefficient | ±2-5% |
| Critical Heat Flux (CHF) | ±5 % |
| Bubble departure frequency (Hz) | ±4% |
| Bubble departure volume ($mm^3$) | ±0.05 $mm^3$ |

Note 3. **Bubble departure diameter and frequency**

Bubble departure frequency and diameter were calculated by averaging the obtained values for at least 10 nucleation sites per case, where 5 sequential bubbles in the images were tracked from growth initiation to the time they reached to the middle of the image frame. Manual pixel-wise calculation were used to determine the locations of diametrical points on bubbles. For each time interval the bubble centroid location was obtained by averaging the diametrical x and y coordinates. When the bubble radial growth becomes constant, time history of vertical position of the bubble centroid approximates the bubble departure frequency. This approximation is in agreement with Rayleigh [3], Mikic, Rohsenow, and Griffith [4].

Due to constant radial growth rate assumption for attached bubble, the centroid location shows a linear change. Straight lined were fitted to the growth and rising portions. The intersection of the growth line with horizon (x-axis), and growth line and rising lines give the initiation and departure points, respectively. The proposed calculations are in parallel with the method recommended by McHale and Garimella [5].

The general governing equation and boundary conditions for the freely rising bubble is given as follow:

$$\begin{aligned} &\ddot{y} + \frac{3}{4}\frac{\rho_l C_D}{\rho_v D}\dot{y}^2 = \frac{(\rho_l - \rho_v)g}{\rho_v} \\ &y(t_0) = y_c\big|_{t=t_0} \\ &\dot{y}(t_0) = \dot{y}_c\big|_{t=t_0} \end{aligned} \quad (6)$$

The first term in the left hand side indicates the acceleration of the rising bubble, the second term in the left hand side are included to inspect the effect of drag force, and the right hand side brings up the importance of buoyancy force effect on rising bubble. Consequently, in equation 6 the drag coefficient of a bubble, time of departure, gravitational acceleration, position and densities of liquid and vapor phases are represented as $C_D$, $t_0$, $g$, $y$, $\rho_l$ and $\rho_v$, respectively. The drag coefficient values in the range of 0.14~1.22 were used in the analysis (according to the proposed values in correlations of Michaelides [6] and Ishii and Zuber [7], respectively). The bubble departure frequency on the bare silicon, and biocoated surfaces with thicknesses of 1µm and 2µm were obtained as (~7.5 Hz, ~33 Hz, and ~38 Hz, respectively. The bubble departure diameters on biocoated surfaces were obtained within the range of 1-3.5 mm size, while the uncoated surface has the departure diameter within the range of 1-2 mm. One of the main reasons for

the difference in bubble departure frequency and departure diameter between biocoated and uncoated surfaces is the presence of porous layer and the bubble departure mechanism prior to the departure process [8].

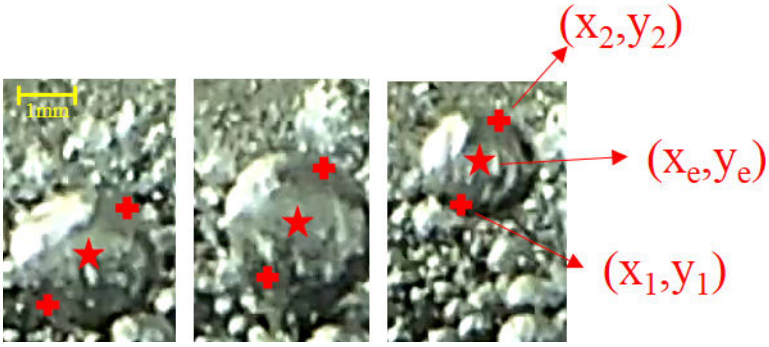


**Supplementary Figure 4.**

Determination of bubble departure frequency and diameter

References


1. Goldstein, J. et al. Scanning electron microscopy and X-ray microanalysis: a text for biologists, materials scientists, and geologists. (Springer Science & Business Media, 2012).
2. Coleman, H.W. & Steele, W.G. Experimentation, validation, and uncertainty analysis for engineers. (John Wiley & Sons, 2009).
3. Rayleigh, L. VIII. On the pressure developed in a liquid during the collapse of a spherical cavity. *The London, Edinburgh, and Dublin Philosophical Magazine and Journal of Science* **34**, 94-98 (1917).
4. Mikic, B., Rohsenow, W. & Griffith, P. On bubble growth rates. *International Journal of Heat and Mass Transfer* **13**, 657-666 (1970).
5. McHale, J.P. & Garimella, S.V. Bubble nucleation characteristics in pool boiling of a wetting liquid on smooth and rough surfaces. *International Journal of Multiphase Flow* **36**, 249-260 (2010).
6. Michaelides, E.E. Hydrodynamic force and heat/mass transfer from particles, bubbles, and drops—the Freeman scholar lecture. *Journal of fluids engineering* **125**, 209-238 (2003).
7. Ishii, M. & Zuber, N. Drag coefficient and relative velocity in bubbly, droplet or particulate flows. *AIChE Journal* **25**, 843-855 (1979).
8. Chien, L.-H. & Webb, R.L. Measurement of bubble dynamics on an enhanced boiling surface. *Experimental Thermal and Fluid Science* **16**, 177-186 (1998).
